# How spirituality is understood in occupational therapy: A qualitative study

**DOI:** 10.1111/1440-1630.70006

**Published:** 2025-03-13

**Authors:** Heather So, Lynette Mackenzie, Chris Chapparo, Judy Ranka, Mary Ann McColl

**Affiliations:** ^1^ Discipline of Occupational Therapy, School of Health Sciences, Faculty of Medicine and Health The University of Sydney Sydney New South Wales Australia; ^2^ The Faculty of Health Sciences Queen's University Kingston Ontario Canada

**Keywords:** Australia, occupational therapy, spirituality

## Abstract

**Introduction:**

Since the inception of occupational therapy, spirituality has been a unique component of practice. However, much of the professional discourse on how to define spirituality has originated internationally. This study aimed to explore how Australian occupational therapists interpret and understand spirituality in their practice.

**Methods:**

This study employed an interpretive phenomenological methodology and reflexive thematic analysis. Twenty‐three individual interviews were conducted with Australian occupational therapists across various work and specialty contexts.

**Consumer and community involvement:**

Because the participants in this study were occupational therapists, the research design did not include input from consumers or the community.

**Findings:**

Three main themes emerged from this study: (i) definitions of spirituality were complex and often described a person's connection to meaning and purpose in life; (ii) human factors that supported addressing spirituality included therapist self‐reflection and clinical experience, while therapist discomfort and specific client circumstances acted as barriers; and (iii) environmental factors that supported addressing spirituality included access to training and a supportive workplace, while barriers included cultural taboos, as well as time and funding limitations.

**Conclusion:**

All occupational therapist participants acknowledged spirituality as the meaningful connection between one's inner self and the outer world, and for most participants, this included acknowledging the transcendent. Therapist skills that facilitated the integration of spirituality into practice included self‐reflection and building rapport with clients, which therapists could control. However, factors like years of clinical experience were beyond their control. In terms of environmental factors, therapists could influence their access to spirituality training, but aspects like a supportive work environment were often outside their influence. Additionally, many therapists reported feeling uncomfortable discussing spirituality within what they considered to be a culturally closed local context. To help overcome these challenges, the occupational therapy profession could benefit from developing culturally sensitive spirituality resources, research, and training.

**PLAIN LANGUAGE SUMMARY:**

Spirituality has always been a part of occupational therapy, but much of the talking about it comes from other countries. This study looked at how Australian occupational therapists see spirituality. We interviewed 23 occupational therapists from all different jobs. They all agreed that spirituality is about connecting a person's inner self with the world. While therapists could control things like self‐reflection and building relationships with clients, their years of clinical experience was outside their control. Likewise, they may be able to look for further education, but a supportive work environment was often out of their hands. Many therapists also felt uncomfortable discussing spirituality in workplaces where it was not openly accepted. To face these challenges, the profession may need more resources, research, and training on spirituality in practice.

**Key Points for Occupational Therapy**

- Spirituality was frequently understood as a person finding connection to meaning and purpose in life.
- Human factors that support spirituality in clinical practice included therapist self‐reflection and clinical experience, whereas therapist discomfort and particular client circumstances were barriers.
- Environmental factors that facilitate spirituality in clinical practice included training and supportive workplaces, whereas cultural taboo and time and funding constraints were significant barriers.

## 1 INTRODUCTION

Early pioneers of occupational therapy, such as Eleanor Clarke Slagle, recognised the ‘spiritual’ dimension of the profession (Bing, 1997, p. 222). Since the 1990s, spirituality has been established as a core part of many occupational therapy models of practice (Chapparo & Ranka, 1997; Townsend, 1997) and has been a key topic in professional discourse (Christiansen, 1997; Egan & Delaat, 1997; Urbanowski & Vargo, 1994). However, much of the conversation on spirituality in occupational therapy has historically been led by countries such as the United States (Hemphill, 2019; Keiter Humbert, 2015), Canada (Beagan & Kumas‐Tan, 2005; Egan, 2000; McColl, 2011), and the United Kingdom (J. Jones et al., 2016; Mayers & Johnston, 2008). Yet, the Australian context is a unique cultural blend of growing secularism, multiculturalism, diverse religions, and indigenous heritage (Australian Human Rights Commission, 2024). There is a growing need for more profession‐specific dialogue about spirituality which is also appropriate for the local context (Best et al., 2022; Fernández & Pulido, 2023).

Spirituality has been defined in an international health care consensus definition as the way ‘persons seek ultimate meaning, purpose, and transcendence, and experience relationship to self, family, others, community, society, nature, and the significant or sacred.’ (Puchalski et al., 2014, p. 646). However, translating such concepts of transcendence, connection, and meaning into clinical practice within the medical model presents numerous challenges. Globally, occupational therapists consistently acknowledge the importance of spirituality but report difficulties in incorporating it into practice (Morris et al., 2014; Mthembu et al., 2017). Barriers such as workplace time pressures (Babaei et al., 2022; Pham et al., 2022), and a lack of questions within workplace documentation (Belcham, 2004; Thompson & Gee, 2018), have been reported by international occupational therapists. Yet, research on the specific barriers faced by Australian occupational therapists in this area remains scarce.

Several factors may help enable an occupational therapist to address a client's spirituality (Keiter Humbert, 2016). For example, a South African occupational therapy Delphi study identified a broad educational base that supports this professional understanding of spirituality, including topics such as history, religion, diversity, coping strategies, injustice, and morality (Mthembu et al., 2018). As well as this knowledge, multiple international occupational therapy studies have also identified workplace supports and professional resources that may help occupational therapists to understand and apply spirituality into practice (Babaei et al., 2022; Egan & Swedersky, 2003; Pham et al., 2022). Despite this range of international research, there is limited primary research of what may help occupational therapists integrate spirituality within the Australian context.

Studies on how Australian occupational therapists understand spirituality in their practice are limited, and existing studies do not explore multiple specialty areas (Leitao et al., 2023). A scoping review of the Australian literature identified only four studies led by occupational therapy authors on spirituality in health care between 2002 and 2022: three were topic reviews, and one was multidisciplinary in nature (So et al., 2023). Therefore, this study aimed to identify how occupational therapists define spirituality and the barriers and facilitators they experience within this context of practice.

## 2 METHODS

### 2.1 Research approach

This study was approved by the Human Research Ethics Committee (HREC) at The University of Sydney, in line with the *National Statement on Ethical Conduct in Human Research (2007)*, with approval number 2022/684. To explore participants' lived experiences of spirituality in occupational therapy, this study adopted an interpretive (hermeneutic) phenomenological methodological approach to guide the study questions and interview approach (Neubauer et al., 2019; Taylor, 2013). Interpretive phenomenology aims for both description and interpretation of the phenomenon, recognising the influence of setting and articulating ‘what is taken for granted’ (Reed, 2024, p. 90). To structure data analysis, Reflexive Thematic Analysis (RTA) (Braun & Clarke, 2021) was utilised, as it provided a systematic yet adaptable approach. Semi‐structured individual interviews were selected to encourage participant self‐expression while maintaining confidentiality. Interviews were conducted over Zoom Video Communications software (Zoom Video Communications, Inc., 2022) to allow maximum accessibility to participants (Gray et al., 2020). Phenomenology combined with semi‐structured interviews and Thematic Analysis has been used in other recent studies (Keten Edis & Kurtgöz, 2024; Landrum et al., 2023) and so was deemed an appropriate approach to explore these sensitive and under‐researched topics.

### 2.2 Consumer and community involvement

Given that the participants of this study were occupational therapists, the research design did not involve consumer and community input.

### 2.3 Positionality statement

The research team aimed to approach the topic with open curiosity, having all previously published research on spirituality in occupational therapy. The research team of this study are occupational therapists, with four based in Australia (HS, LM, CC, and JR) and one in Canada (MAM). This research is part of HS's PhD candidature, while the other members (LM, CC, JR, and MAM) are late career academics. HS initiated the research based on familial and clinical encounters with spirituality in health‐care settings.

### 2.4 Recruitment strategy

The recruitment strategy targeted participants who were Australian occupational therapists. To ensure a diverse group, maximum variation sampling was engaged (Douglas, 2022). Recruitment materials were shared via informal email networks targeted at occupational therapists and posted in occupational therapy Facebook groups.

Interested participants were asked to complete an expression of interest survey, which included demographic questions and a confirmation of current registration with the Australian Health Practitioner Regulation Agency (AHPRA). This resulted in 30 completed expressions of interest, along with three incomplete submissions. All participants who expressed interest were provided with a Participant Information Form and a Participant Consent Form. Written consent was obtained from all participants who agreed to take part.

In total, 23 participants confirmed their willingness to participate in interviews. The sample size for the study was primarily determined by the availability of participants, as well as the time and resources available to conduct the interviews. The interview schedule was sent to participants the day before the interview to assist with their preparation. On the day of the interview, a verbal summary of the consent process was provided to ensure understanding. Furthermore, all participants accepted the offer of being provided with feedback on the overall study results.

### 2.5 Data collection

The interview schedule is included in Table 1. In this interview schedule, and in the demographic specialty area 'General OT' used throughout, the abbreviation 'OT' means 'occupational therapist'. HS conducted all interviews via Zoom. All participants participated in one interview each, lasting approximately 1 h. HS also made consistent notes of any preliminary analysis (Nowell et al., 2017). Interviews were audio and video taped via Zoom Video Communications software. Video data were promptly deleted post interview, to further protect participant anonymity. HS transcribed the audio recordings verbatim, ensuring that participant transcripts were de‐identified and assigned unique pseudonyms at the time of transcription. The University of Sydney has appropriately stored all data.

**TABLE 1:** Interview schedule.

Topic	Interview question
*Background understanding*	Please tell me briefly about your current role as an OT.
Definitions	How you define spirituality? How do you differentiate that from religion and culture?
Barriers	What do you think are the barriers to OTs addressing a client's spirituality?
Facilitators	What do you think would help OTs to address a client's spirituality in practice?
*Background understanding*	Do you have anything else you would like to add?

### 2.6 Data analysis

The participants were given the opportunity to review and clarify their interview transcripts to enhance the research findings (Motulsky, 2021). Ten participants availed themselves of this opportunity. See Table 2 for details of the application of RTA to the participant interview data.

**TABLE 2:** Application of reflexive thematic analysis (RTA).

Phase of RTA	Details
*Phase 1:* *familiarising yourself with the dataset*	HS became deeply familiar with the data by manually transcribing all interviews. Each interview was listened through multiple times by HS.
*Phase 2: coding*	HS and LM independently hand‐coded three initial interviews and collaboratively developed a preliminary codebook To establish an audit trail, all discussions between the research team were documented.
*Phase 3: generating initial themes*	The interviews were coded independently in small batches, with regular comparisons and refinements of the codes. The two sets of coded interviews were then combined and organised within NVivo Qualitative Data Analysis Software (QRS International, 2020). This helped ensure rigour, as all codes were systematically catalogued.
*Phase 4: developing and reviewing themes*	The trustworthiness of the findings was ensured through triangulation, with feedback on the themes and their clarity provided by CC, JR, and MAM and three peer occupational therapists Throughout the process, the researchers engaged in self‐reflexivity, regularly reflecting on their own responses to the topics raised by the participants
*Phase 5: refining, defining, and naming themes*	All researchers reviewed the themes and the overall presentation of the findings, offering edits and feedback until an agreement was reached.
*Phase 6: writing up*	A report was produced, including detailed findings. CC, JR, and MAM individually edited the final article, with HS and LM working collaboratively to integrate each researcher's feedback.

## 3 FINDINGS

### 3.1 Participants demographics

A total of 23 participants were interviewed. Most were female (91.3%, *n* = 21). Overall, many participants had significant working experience. One participant identified as having 0–1 years working experience (4.3%), three had 5–10 years working experience (13.0%), 14 had 10–20 years working experience (60.1%), four had 20–30 years working experience (17.3%) and one had 30+ years working experience (4.3%). The state and territory location of participants was predominantly on the east coast of Australia, and included NSW (65.2%, n = 15), QLD (17.4%, n = 4), Vic (21.7%, n = 3), and WA (4.3%, n = 1). There was a wide range of specialties represented, which included paediatrics (6/26.1%), aged care and rehabilitation (4/17.4%), palliative care and oncology (3/13%), acute and emergency (3/13%), mental health (2/8.7%), adult disability (1/4.3%), general OT, all ages (2/8.7%), care co‐ordination (1/4.3%), and women and perinatal health (1/4.3%). Finally, self‐reported religious or spiritual affiliation included Christian or Christian denomination (11/47.8%); NA, Nil, or ‘no label’ (9/39.1%); and ‘Other’ (3/13%).

### 3.2 Themes

The findings are organised around the three main themes identified from the interviews: (i) the participants' definitions of spirituality, (ii) human factors that influenced the integration of spirituality in practice, and (iii) environmental factors that affected addressing spirituality in practice. The participant pseudonym numbers are used, denoted by a ‘P,’ along with their occupational therapy specialty area to provide background for the quotes.

#### 3.2.1 Theme 1: Spirituality as connection

**FIGURE 1:**
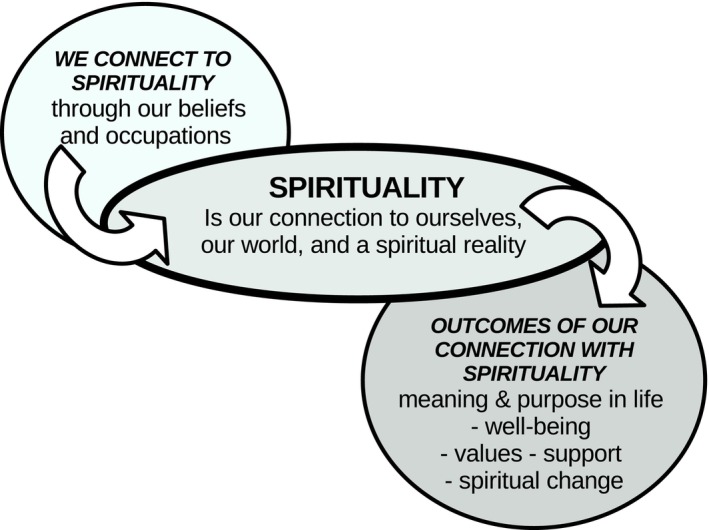
Spirituality concepts in occupational therapist participant definitions.

The participants were asked to define spirituality, and their responses were organised conceptually in Figure 1. The participants frequently defined spirituality as a person connecting to themselves, and to the physical and spiritual world, which may lead to a variety of outcomes. Overall, most participants reported that defining spirituality was difficult, and almost all participants stated that this was the first time that they had tried to define these terms in a professional setting.

##### We connect to spirituality through our beliefs and occupations

Every participant described spirituality as being person‐centred and involving connections both within oneself and with the outside world. Spirituality was understood through personal beliefs, particularly those linked to existential questions such as: ‘Who am I?’ (P18, Aged Care and Rehabilitation); ‘What am I doing in my life?’ (P6, Mental Health); and ‘Why do things happen? Is there a power out there that's bigger than me that it's making these things happen?… How do I fit in? How am I going to make a difference?” (P21; Acute and Emergency). Spirituality was also expressed through engagement in various meaningful occupations, such as ‘ritual’ (P11, Acute and Emergency), ‘practices’ (P15, Palliative Care and Oncology), and ‘experiences’ (P18, Aged Care and Rehabilitation). These occupations could be secular, for example, ‘I enjoy being in nature … and just connecting to others’ (P3, Paediatrics), or spiritual/religious, for example, ‘it could be yoga … reading spiritual literature … people can pray or meditate or chant … they could attend church or synagogue’ (P14, Adult Disability). In this way, the participants described spirituality as encapsulated in existential beliefs and expressed through a range of meaningful occupations.

##### Spirituality is our connection to ourselves, our world and a spiritual reality

Many participants spoke about spirituality as being linked to the concept of having a spirit. One participant noted, ‘your spirit is a deep part of who you are, and so spirituality has to do with that deep part of who you’ (P1, Aged Care and Rehabilitation). A person's spirit was often described as their ‘inner essence’ (P7, Women's and Perinatal Health) which ‘transcends your physical being’ (P17, Aged Care and Rehabilitation). Most participants described spirituality as a person ‘being connected to something that's greater than yourself as an individual’ (P4, Paediatrics). Many also emphasised the community aspects of spirituality, including connections with ‘the world around you and the people in the environment’ (P16, Palliative Care and Oncology). For one participant, this meant ‘connecting to the land’ and recognising the importance of relationships with ‘elders who have come before us’ (P22, General OT). Many participants described spirituality as a connection to a spiritual or transcendent reality, which they described as a ‘spiritual realm’ (P22, General OT) or ‘environment’ (P13, Mental Health). For some, this involved ‘thinking of a higher power or order to rely upon, guiding life,’ which may include ‘God’ (P9, Care Coordination). Overall, the participants described spirituality as a deep connection to oneself, and to relationships with others, the environment, and a spiritual (or transcendent) reality.

##### The outcomes of connecting with spirituality

The outcomes of connecting with spirituality were largely viewed as positive, although some participants noted that the process could also be challenging for individuals. Most participants described spirituality as a means of finding ‘purpose in life’ (P14, Adult Disability) and ‘creating meaning of what life is all about for you’. (P8, Acute and Emergency). Some participants spoke about how spirituality helped individuals develop deeply held values. The ‘well‐being’ outcomes of spirituality included ‘finding a sense of fulfilment’ (P14, Adult Disability) and ‘sources of hope for that person or family through challenging times’ (P2, Paediatrics). Some participants also noted that spirituality could lead to ‘progression and growth’ (P23, General). The positive outcomes of spiritual connection were also evident within communities, including religious communities. As one participant noted, ‘there's so much that comes into that formal religion as well … it is that sense of community. It is a way that people bond with their families and have something in common’ (P21, Acute and Emergency). Overall, the participants described that connecting with spirituality was generally a positive experience, offering individuals a sense of meaning, purpose, hope, personal growth, and community bonds.

##### Religion and culture in relation to spirituality

Most participants described both religion and culture as concepts focussed on group connection. Religion was frequently described as groups connected around ‘a common belief system’ (P4, Paediatrics), whereas culture was often defined as being connected to the people around you and your past. Religion seemed to be a more emotionally charged topic for many participants, with some expressing conflicting feelings about it, particularly about how religion fit within the local secularised context. For instance, some participants associated religion with ‘empty ritual,’ noting that ‘some people may participate in religious rituals, but … it's not a deep part of who they are’ (P1, Aged Care and Rehabilitation). Others reflected on the misuses of religion, citing personal experiences with cults or the historical role of religion in the colonisation of Australia. Some participants who identified with a particular religion said that they preferred to describe themselves as ‘having faith’ rather than ‘being religious’. Additionally, some participants reflected on culture in relation to the specific considerations involved in clinical practice with different cultural groups, including Aboriginal and Torres Strait Islander Peoples. For these participants, discussions about spirituality in relation to religion and culture appeared to be strongly shaped by their personal experiences and the local context.

##### Facilitators and barriers: human and environmental factors

**FIGURE 2:**
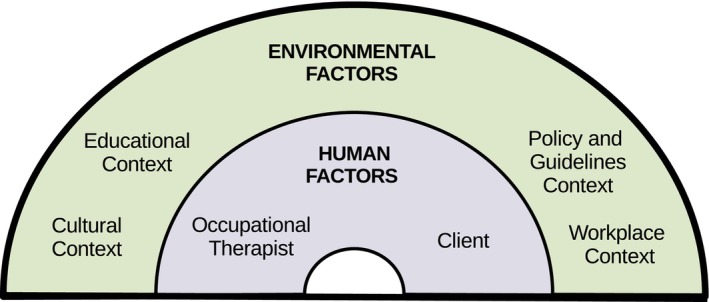
Human and environmental factors that affect addressing client spirituality in clinical practice.

The participants were asked about barriers for, and what would help, occupational therapists in addressing client spirituality in practice. Issues relating to barriers and facilitators for integrating spirituality into practice were organised into human and environmental factors, as seen in Figure 2. Factors were considered human if they related to the occupational therapist or the client and are explored in Theme 2. Factors were considered environmental when they related to the cultural context, policy and guideline, educational, or workplace context, and are explored in Theme 3.

#### 3.2.2 Theme 2: The ebb and flow of interactions between clients and therapists around spirituality

The two most reported human factors that facilitated a therapist addressing client spirituality was a meaningful personal experience with spirituality and increased clinical experience. A meaningful personal experience with spirituality was described as arising from self‐reflection during difficult times, such as a therapist dealing with ‘burnout’ (P21, Acute and Emergency) and ‘grief and loss’ (P11, Acute and Emergency). One participant noted that when an occupational therapist has ‘encountered some hardship or struggle … they've had to search within themselves, and to cope with something, and it makes them more … compassionate and aware of the difficulties in other people's lives” (P19, Palliative Care and Oncology). In this way, the participants reported that self‐reflection during challenging times enhanced their consideration of their clients' spirituality.

Additionally, having clinical experience and confidence in justifying spirituality as part of the occupational therapy role was also seen as helpful in addressing client spirituality. As one participant explained, ‘now I'm a more senior clinician, I have … the courage and the competence to be able to justify why I'm working in that [spiritual] area’ (P21, Acute and Emergency). Clinical experience also contributed to a deeper understanding of how spirituality can manifest for clients, including being expressed non‐verbally. One participant shared, ‘I think it's about observation … sometimes I'll just ask a kid in the assessment like, “What do you like?” … And I really observe their response to that … I think if you have a client, that kind of just shrugs like, *“I'm not connected to anything”* … that's great information’ (P4, Paediatrics). In this way, the participant was describing their development of clinical expertise in using informal spirituality questions, recognising both verbal and non‐verbal expressions of client spirituality, and their comfortability with client negative responses.

The human factors that made addressing client spirituality more challenging were an occupational therapist's personal discomfort with spirituality, and increased caution when working with clients in particular circumstances. Difficult emotions expressed by participants included, ‘we're scared to talk about it … it can be seen as an uncomfortable conversation’ to have with clients (P3, Paediatrics). Additionally, some participants reported that their workplace leaders also appeared uncomfortable, so ‘we just avoid it because it's hard … [and] a lot of supervisors also wouldn't be comfortable to go there’ (P13, Mental Health). This barrier was further compounded by concerns about how to adequately address client spiritual concerns if they arose, with one participant wondering, ‘what if I open up this can of worms, then what am I gonna do?’ (P8, Acute and Emergency). Fear of awkward conversations, lack of workplace leadership, and uncertainty about how to address client spiritual concerns were a common barrier.

Furthermore, specific client groups were considered particularly vulnerable or having unique communication needs, which made approaching spirituality more complex for therapists. For example, with children ‘spirituality is a lot to unpack for paediatrics’ (P4, Paediatrics); and in mental health settings, where one participant noted, ‘I've seen on top of their PTSD, it can be really dangerous for them to go down that spirituality track’ (P6, Mental Health). As one therapist who had multiple clients with complex family situations summarised, ‘sometimes it's very hard to approach’ (P20, Paediatrics).

#### 3.2.3 Theme 3: The environmental conditions that affect addressing spirituality in practice

The two most important environmental factors that facilitated addressing spirituality were educational training or resources, and a supportive workplace. Nearly all participants identified that spirituality training and resources, including culturally sensitive approaches and diverse learning platforms, would be a key environmental factor influencing their ability to address spirituality in practice. Many expressed the need for educational opportunities that provided ‘culturally appropriate ways to talk about spirituality, ways to pose those questions that won't necessarily trigger people’ (P1, Aged Care and Rehabilitation) within a local context that many participants considered closed to spiritual conversations. The participants also highlighted the importance of having a variety of learning platforms, such as ‘webinars, podcasts … visual resources … strategies on how to ask the question about people's spirituality and … strategies around how to respond’ (P3, Paediatrics).

Moreover, a spiritually supportive workplace was also considered essential by many for supporting client spirituality. Such workplaces were diverse and included ‘an organization with a faith background’ (P10, Aged Care and Rehabilitation), ‘private practice’ (P6, Mental Health), longer stay wards such as ‘rehab’ (P21, Acute and Emergency), and culturally specific services for Aboriginal and Torres Strait Islander Peoples. The aspect of a workplace which made it supportive was described by one participant as ‘they naturally accept that you can talk about [spiritual] things’ and spirituality is ‘in the assessment process’ (P10, Aged Care and Rehabilitation). Additionally, four participants explicitly stated that they had left government hospital positions to move to community‐based workplaces that allowed for a more holistic approach to client care, including addressing client spirituality. So, whether faith‐based, long‐stay, private practice, culturally specific, or community‐based, a ‘supportive’ workplace was reported as helpful for addressing client spirituality.

However, the key environmental barriers to spirituality was the cultural context, and time and funding constraints. The cultural context, both within occupational therapy and broader society, was frequently reported by participants as being closed to spiritual or religious conversations. One participant noted, ‘spirituality is a taboo topic in OT, and generally in Australian society. If it isn't a particular way or form of spirituality that's more culturally accepted … there's a big focus on ‘wellness initiatives’ … it's challenging when I guess my own understanding of spiritualities is beyond that. But even just discussing that can come across as offensive or disrespectful, or “this is not welcomed”’ (P9, Care Coordination). Many participants also reported versions of the saying, ‘don't talk politics, don't talk religion … in Australian culture’ (P11, Acute and Emergency). Workplace and professional environments also often ignored spirituality. One participant explained, ‘there's often cultural things and … sexuality [training and guidelines] and all that kind of stuff. But I don't think that there was anything particularly about spirituality’ (P1, Aged Care and Rehabilitation). In this way, spirituality and religion were described by participants as taboo topics both within healthcare contexts and the local cultural context.

Likewise, a significant environmental barrier to addressing client spirituality was the lack of time or funding, especially for those working in an ‘acute hospital setting’ (P15, Palliative Care and Oncology) or under limited‐hours funding. As one participant commented, ‘we don't have time to really explore it with them, even if people bring it up’ (P17, Aged Care and Rehabilitation). Additionally, lack of ‘adequate staffing’ (P19, Palliative Care and Oncology) and therapists being referred to see a client ‘the day of discharge’ (P11, Acute and Emergency) also added time pressures that created barriers to addressing client spirituality. Cultural and practical restraints were reported as the main environmental barriers to occupational therapists addressing client spirituality.

## 4 DISCUSSION

The occupational therapists in this study reported rich and nuanced understandings of spirituality and its associated concepts, aligning with findings from other HP studies (Paal et al., 2023; Shah et al., 2018). Several of the ways that participants defined spirituality, such as the connection between spirituality and key relationships influencing outcomes, reflect themes found in recent theoretical work (Cao et al., 2020; de Brito Sena et al., 2021). However, many participants indicated that this was the first time in their careers that they had actively reflected on spirituality in a professional context, making the conversation feel unfamiliar. This finding echoes results from other international occupational therapy studies (Morris et al., 2014; Mthembu et al., 2015), which highlighted a gap between the theoretical understanding of the significance of spirituality and its practical application in clinical settings.

While the definitions of spirituality used by participants shared significant overlap with the widely accepted consensus definition (Puchalski et al., 2014), they also expanded on it in two key ways. First, many participants framed spirituality through the lens of individual beliefs about existential questions, an approach also utilised by Australian chaplains (Carey et al., 2018). Second, many participants included the idea of a spiritual realm or higher power in their definition of spirituality, a transpersonal aspect recognised in a prominent occupational therapy definition (Johnston & Mayers, 2005) and also by many indigenous and religious approaches to spirituality (Eyres et al., 2019; Grieves, 2009). In summary, the participants' definitions of spirituality not only mirrored established views but also introduced additional layers related to existential beliefs and the presence of a spiritual reality or higher power, appropriate to the local context.

Religion and culture were described as group‐based concepts that may or may not overlap with an individual's spirituality (Arrey et al., 2016; Jensen, 2021). However, most participants found it difficult or uncomfortable to define these concepts, a challenge also reported by Australian paediatric occupational therapists (Leitao et al., 2023). This may partly reflect Australia's cultural and religious history, including the connection of Christianity with British colonisation (Rademaker, 2016) and recent trends of increasing secularisation (Australian Bureau of Statistics, 27 June, 2017). While cultural consideration, particularly of Aboriginal and Torres Strait Islander Peoples, has been clearly articulated for occupational therapy practice (AHPRA & National Boards, 2022), there are fewer professional guidelines relating to religion. Despite the challenges, considering a client's religion and culture remains essential in addressing diversity within occupational therapy practice (Passmore, 2003; Pentaris & Thomsen, 2020).

In Canada, occupational therapists have similarly reported the benefits of years of clinical experience, with ‘spiritual considerations … developed gradually after [occupational therapists] began to feel more comfortable with more tangible areas of therapy’ (Egan & Swedersky, 2003, p. 530). Additionally, the process of becoming aware of and managing one's own beliefs in client interactions has also been explored within British occupational therapy (Belcham, 2004; Collins, 2007). These self‐reflection skills can be strengthened through professional development activities (Barry & Gibbens, 2011). Although years of clinical experience may be beyond an occupational therapist's control, promoting a professional environment that encourages self‐reflection on personal beliefs is within their ability to influence.

Uncomfortable personal feelings and a closed cultural context were identified as significant barriers for occupational therapists in addressing spirituality. Similar experienced feelings of local unease have been reported by Australian nurses (Keall et al., 2013) and rehabilitation HPs (K. Jones et al., 2020). These findings underscore the need for a clear and validated articulation of the role of spirituality in health care, particularly within workplace administration and assessment documents (Gardner, 2020). Addressing such cultural barriers is crucial for integrating spirituality into health care, with clear policies and documentation needed to support this practice.

Training, whether at the undergraduate or postgraduate level, was recognised as a key environmental facilitator that would help therapists to address spirituality with clients. A similar need for both types of training has been reported by Australian social workers and doctors (Gardner, 2020; Rombola, 2019), as well as occupational therapists in the United States, Canada, Iran, and South Africa (Babaei et al., 2022; Egan & Swedersky, 2003; Leitao et al., 2023; Morris et al., 2014; Mthembu et al., 2015). It appears that there is a widespread need for spirituality in health care to be more explicitly taught across professions worldwide. Similar to other recent Australian HP studies (Best et al., 2022), the participants in this study also emphasised that such training should be tailored to address local sensitivities around spiritual, religious, and cultural discussions. Additionally, many of these participants also emphasised the need of practical spirituality training and stories that related directly to typical clinical scenarios (NHS Education for Scotland, 2021).

Workplace factors play a crucial role in supporting client spirituality to be addressed. Internationally, spiritually supportive workplaces have been described by occupational therapists as those outside of hospital settings (Hess & Ramugondo, 2014), or those where spiritual or religious practices were incorporated into the workplace evaluation processes (Thompson & Gee, 2018). The participants in this study also noted environmental challenges such as limited time and funding within the health system. These workplace barriers have been widely acknowledged by other HPs, including doctors, nurses, and social workers (Estacio et al., 2018; Hegarty et al., 2005; Lynn & Mensinga, 2015). Spirituality is a delicate and sometimes private topic within the Australian context, and without the appropriate time given to clients, these important topics may not be able to reach the surface in therapeutic encounters (Best et al., 2024). In summary, workplace factors—both supportive and limiting—significantly impact on an occupational therapist's ability to address spirituality in clinical practice.

### 4.1 Recommendations

All participants described having a multifaceted understanding of spirituality and recognised its value. However, they also highlighted the challenges in health‐care settings that rarely support the integration of spirituality into clinical practice. The participants suggested several structural changes to develop the professional discourse around spirituality, including enhanced training opportunities, opportunities for self‐reflection on personal spiritual experiences, the creation of more spirituality‐inclusive workplaces with clear guidelines and assessment tools, as well as dedicated time and funding to address spiritual concerns. Occupational therapists could benefit from increased professional support to enhance their understanding and ability to address spirituality in clinical practice.

### 4.2 Limitations

The findings generated by this study were obtained from a small sample of voluntary participants, reflecting possible response bias. Additionally, the participants in the geographical areas on the east coast of Australia were dominant, and only the states and territories of NSW, QLD, Vic, and WA were represented in this study. There was also a lack of some minority spiritual and religiously affiliated participants in this study. To validate findings, it may be beneficial to survey a broader range of occupational therapists to reach a group of participants who would reflect a more diverse perspective of spirituality in occupational therapy practice (Braun et al., 2021). Additionally, in line with study aims, culture and religion were only explored in this study in relation to spirituality, not as discrete concepts, which may have limited the exploration of these concepts.

## 5 CONCLUSION

All participants recognised spirituality as the link between an individual's inner spirit and the external world, with many also recognising a transcendent aspect to life. Therapists could control developing skills like self‐reflection and building rapport with clients to help integrate spirituality into their practice. However, factors such as years of clinical experience were beyond their control. Regarding environmental factors, therapists could influence their access to spirituality training, but a supportive work environment was often outside their influence. Additionally, many therapists felt uneasy discussing spirituality in what they perceived as a culturally restrictive local context. To address these challenges, the occupational therapy profession could benefit from developing culturally sensitive resources for integrating spirituality into practice.

## AUTHOR CONTRIBUTIONS

All authors contributed to the study conception and design. Study design, data collection, data analysis, and manuscript writing were performed by HS. Initial code book development was conducted by HS and LM. LM provided primary supervisory support, manuscript editing, validation of the data collection, analysis, and results presentation. CC and JR assisted in the data analysis and manuscript editing. MAM provided manuscript editing and content supervision. All authors edited the results presentation and themes developed. All authors approved the final manuscript.

## CONFLICT OF INTEREST STATEMENT

‘The authors have no relevant financial or non‐financial interests or conflicts of interest to disclose.’

## ETHICS APPROVAL

The ethical aspects of this study were approved by the Human Research Ethics Committee (HREC) of The University of Sydney according to the National Statement on Ethical Conduct in Human Research (2007). Approval number 2022/684.

All potential participants received a Participant Information Form and Participant Consent Form and were asked to complete written consent. On the day of the interview, the consent summary for the study was again provided to participants verbally.

All participants gave their consent to publish.

## Data Availability

Research data are not shared. The data used in this study are considered highly sensitive and connot be shared, in line with the approval conditions of the Human Research Ethics Committee (HREC) of the University of Sydney.
